# Impact of dualism on the perception of treatability in psychiatry

**DOI:** 10.5339/qmj.2025.34

**Published:** 2025-06-09

**Authors:** Javed Latoo, Minal Mistry, Majid Alabdulla, Farida Jan, Sheikh Mohammed Shariful Islam, Yousaf Iqbal, Tom Howseman, David Riley, Tariq Munshi, Mudasir Firdosi, Daljit Sura, Ovais Wadoo

**Affiliations:** 1 Department of Psychiatry, Hamad Medical Corporation, Qatar; 2 College of Medicine, Qatar University, Doha, Qatar; 3 Department of Psychiatry, Eastern Health, Fredericton, NL, Canada; 4 Department of Psychiatry, Northamptonshire Healthcare NHS Foundation Trust, Northampton, UK; 5 National Health and Medical Research Council, and Institute for Physical Activity and Nutrition (IPAN), Deakin University Melbourne, Melbourne, VIC, Australia; 6 St Luke’s Primary Care Centre, Northamptonshire, UK; 7 Department of Palliative Care, Northamptonshire Healthcare NHS Foundation Trust, Northampton, UK; 8 Department of Psychiatry, University of Toronto, Toronto, ON, Canada; 9 Kent and Medway NHS & Social Care Partnership Trust, Kent, UK; 10 North Street Medical Care, Romford, Greater London, UK *Email: OWadoo@hamad.qa

**Keywords:** Dualism, treatability, treatment approach, mental health, physical health

## Abstract

**Background:** A false division between mental and physical disorders is supported by dualism, contributing to mental health stigma. There is a widespread misconception about the prognosis and treatment options for psychiatric diseases. This is despite data supporting the effectiveness of psychiatric treatments for a variety of illnesses that have been proven by meta-analysis. In general, the efficacy of drugs used to treat physical problems and psychiatric disorders is comparable.

**Methods:** In this article, experts from a variety of fields—including psychiatry, primary care, and general medicine—highlight how the paradigms based on dualism play a crucial role in maintaining the myths regarding psychiatric disorders, particularly those that relate to their treatability in comparison to physical health conditions.

**Results:** There are numerous similarities between mental and physical problems in terms of the causes andtreatment. Healthcare, like other complex human systems, is rife with uncertainty. In actuality, the severity and treatability of both physical and mental diseases range widely. Treatment response varies from person to person. There are certain physical and mental health disorders that respond well to treatment, some that do not, and some for which there are currently no effective cures.

**Conclusion:** We believe that dualism, which promotes the separation of mental and physical phenomena, is the core driving force behind these misconceptions. These fallacies, in our opinion, are primarily motivated by dualism, which advocates the division of mental from physical occurrences.

## INTRODUCTION

Every year, one in four people around the world suffer from mental illness.^1^ Diabetes, heart failure, myocardial infarction, and stroke are among physical conditions that can raise your risk of developing mental health problems.^2^ Individuals who have mental illness, on the other hand, are more likely to develop physical health issues such as cardiovascular disease, respiratory disorders, diabetes, cancer, and infections.^3^ Compared to the general population, those with mental illnesses have more than double the all-cause death rate and die 10–20 years earlier.^3,4^

People with mental illness have excess mortality mainly due to noncommunicable diseases, driven by lifestyle, healthcare inequities, and socioeconomic factors.^3^ The stigma attached to mental health conditions such as anxiety, depression, schizophrenia, and bipolar illness sometimes causes obstacles to receiving help. Healthcare professionals may contribute to this stigma by their unfavorable views, therapeutic pessimism, insufficient training, and a culture that does not value mental health.^5^ As a result, people may be reluctant to seek help, have their treatment terminated prematurely, have a poor patient-provider connection, and be concerned about their safety.^5^

Stigma delays treatment and discourages engagement with available services despite the availability of effective and affordable treatments like antidepressants, antipsychotics, mood stabilizers, and cognitive behavioral therapy. A common misconception is that mental illness differs fundamentally from physical illness, with the former often seen as chronic, incurable, and untreatable. This misperception creates a vicious cycle where increased stigma leads to greater barriers to care, worsened clinical outcomes, and a continued belief in the chronic nature of mental illness.

Dualism—the notion that separates mental and physical phenomena—is a key factor in reinforcing these misconceptions. By creating an artificial divide between mental and physical health, dualism exacerbates the stigma and misconceptions surrounding psychiatric disorders. It influences perceptions of mental health by framing the mind and body as separate entities. This separation can lead people to view mental health issues as abstract or intangible, unlike physical ailments, which are often seen as more concrete. For example, conditions like depression or anxiety might be perceived as matters of willpower or personal weakness, rather than biological or neurological disorders. In healthcare, dualism may contribute to fragmented treatment approaches, where physical symptoms are prioritized, and mental health is overlooked or inadequately addressed, perpetuating gaps in holistic patient care.

In this article, experts from diverse specialties, including psychiatry, primary care, and general medicine, with experience from Australia, Canada, Qatar, and the United Kingdom, explore how dualism perpetuates misunderstandings about mental health. They emphasize how this separation influences perceptions of psychiatric disorders and their comparability to physical health conditions, highlighting the need for a more integrated approach to address these issues.

## ROLE OF DUALISM AND TREATABILITY

### Curability in medicine

A cure generally means complete restoration of health, whereas treatment refers to methods that improve health without guaranteeing complete recovery.^6^ For a treatment to be considered curative, it should alleviate symptoms by addressing the underlying cause, have no side effects, not lead to recurrence after discontinuation, and result in overall health benefits.^7^ The notion of curability in medicine is still controversial despite these standards. According to Pizzorno, a lot of doctors who went into medicine because they wanted to heal people experience cognitive dissonance when they learn that most traditional medications merely treat symptoms rather than the underlying cause.^7^

Medical professionals often use medications, therapies, surgeries, and other treatments to lessen symptoms and improve quality of life. Such treatments can be considered cures if they eradicate the disease. For instance, bacterial infections can be cured with antibiotics. However, many diseases, both physical and mental, are incurable, and treatments focus on controlling symptoms without curing the condition. Diseases like diabetes, hepatitis B, Parkinson’s disease, and schizophrenia are examples of incurable conditions.

### Treatment of incurable and chronic physical health conditions

Various medical conditions—including advanced cancers, AIDS, systemic lupus erythematosus, Crohn’s disease, ulcerative colitis, Parkinson’s disease, multiple sclerosis, sarcoidosis, pulmonary fibrosis, diabetes mellitus, hypertension, arthritis, and chronic liver and kidney disease—are chronic in nature and incurable.^8,9^ In the United States, 60% of adults live with a chronic physical condition, and 40% of Americans have two or more chronic conditions.^10^ Chronic conditions are defined by their persistence for at least 1 year, the necessity for ongoing medical interventions, and their impact on activities of daily living (ADL).^11^ The primary aim of treatment for these conditions is symptom management rather than a complete cure.^12^

Healthcare practitioners often take a proactive approach to managing these chronic illnesses, even though there is not a permanent solution. Therapeutic objectives that doctors set include minimizing symptoms, lowering complications, lowering hospital admissions, and preserving community functioning.^13^ The aim of treatments is to improve patients’ overall quality of life by improving their capacity to do instrumental activities of daily living (IADL), which includes cooking, cleaning, laundry, transportation, and managing finances.^14^ Despite incurability, there is ample evidence of society’s approval and support for this method.^15^

However, it is worth considering whether a comparable method is used for persistent mental health disorders. Despite the chronic nature of physical and mental health issues, treatment techniques may differ. For example, whereas empirical evidence and case studies demonstrate proactive treatment options for physical health conditions,^16^ similar approaches to mental health are frequently overlooked.^17^ More research could help to better understand and improve societal attitudes regarding chronic mental health disorders.^18,19^

### Chronic mental health conditions: a different treatment approach?

There is a common belief that there are substantial differences in the fundamental nature of physical and mental health conditions—but there are surprisingly many similarities. Mental health conditions such as schizophrenia, bipolar disorder, depression, and obsessive-compulsive disorder are chronic as they last a year or more, need ongoing medical care, and impact ADL/IADL. Mental illness, like physical illness, can be acute or chronic, treatable or untreatable, and curable or incurable.^20^ They both may need treatment for the short or long term and, may need more than one treatment and may become treatment-resistant.^20^

However, despite these similarities, healthcare professionals appear to adopt a different approach to treating mental health conditions, as illustrated by several concerning statistics. Mental health conditions often remain undetected and untreated for years due to barriers to accessing care associated with the stigma of mental illness. Most people with a mental illness within 12 months had no treatment, especially in less-developed countries.^21^ This is complicated by delays in seeking treatment—up to 30 years for anxiety disorders, 14 years for mood disorders, and 18 years for substance use disorders.^22^

Healthcare professionals and the public often fail to show the same passion and motivation to achieve similar therapeutic goals for mental illness, such as minimization of symptoms, fewer complications, fewer hospital admissions, ability to maintain functioning in the community, ability to perform ADL/IADL and improvement in quality of life. This may be accompanied by a debate about whether mental illnesses exist or are treatable, thus reinforcing the belief that they are fundamentally different, or even inferior, to physical illnesses and thus deserve less attention. This reinforces the stigma of mental illness and impacts the sufferer’s willingness to seek help or engage in treatment.^23^

The effectiveness of psychiatric treatment is well known and broadly comparable to medications used for physical health, as evidenced by meta-analyses demonstrating the use of antidepressants, antipsychotics, mood stabilizers, and cognitive behavioral therapy in a range of conditions such as major depression, anxiety disorders, bipolar disorder, and schizophrenia.^24–29^ Despite the above-established evidence, the public and healthcare professionals tend to negatively perceive psychiatric treatments and their efficacy. We believe this bias falls largely under the shadow of a dualistic mental framework.

### Dualism and mental health

Dualism, which separated the world into two categories of autonomous substances—the mental and the physical—was first proposed by the French philosopher René Descartes in the seventeenth century.^29^ Descartes maintained that because physical substances and mental processes are inherently distinct from one another and do not interact, physical substances like the brain cannot produce mental processes.^30^ This split created a stigmatizing divide between the mind and the brain, which resulted in the division of psychiatry and neurology and in the more general division of mental and physical health.^20,30^

By leaving the brain domain under the shadow of dualism, psychiatry aligned itself with nonmedical fields such as psychoanalysis. Although this alignment was beneficial in certain aspects, it also provided room for conjecture and involvement from nonmedical individuals such as celebrities, quacks, priests, and philosophers. Dualism placed doubt on the causal relationship between the mind and the brain by promoting a mental framework based on the idea that mental disease could not be explained by brain function. As such, this viewpoint also called into doubt the efficacy of biological psychiatric interventions. This parallels alternative medicine in physical health, which at times promotes pseudoscience and misinformation. Both fields face the shared challenge of embracing innovative ideas while upholding rigorous, evidence-based practices to preserve credibility and ensure the delivery of optimal patient care in mental and physical health.

Unfortunately, despite the lack of support from contemporary neuroscientific research, dualism remains a pervasive influence. Studies among students, healthcare workers, mental healthcare workers, and laypeople demonstrate that many continue to perceive the brain and mind as separate, independent, and unrelated entities.^31–33^ This persistent dualistic thinking is evident even among mental health professionals, who often invoke a mind-brain division in clinical discussions despite formally adopting the biopsychosocial model.^32,34^

The impact of dualism extends beyond philosophical and historical contexts to include practical implications on modern provider attitudes and healthcare practices.^34^ Because mental and physical health are now separate, there is fragmented care, and mental health is frequently ignored or viewed as secondary to physical health.^35^ Numerous factors contribute to this fragmentation, including the ongoing stigma surrounding mental illness,^36^ the underfunding of mental health services,^37^ and the unwillingness of certain healthcare professionals to completely integrate mental and physical health treatment.^38^

Additionally, due to the belief that the mind and brain are separate, dualistic thinking can also make medical professionals reluctant to explore biological treatments for mental health issues.^39^ The overemphasis on psychosocial therapies at the expense of investigating all-encompassing, biologically based treatment approaches may also be a result of this bias.^40^ Consequently, patients could have insufficient treatment that does not fully address the intricacy of their condition.^41^

In addition to ongoing attempts to refute dualistic thinking in healthcare education and practice, increasing integration of neuroscientific insights into clinical practice is needed to address these issues.^35^ Further research demonstrating the connection between physical and mental health can reinforce this integration and contribute to the deconstruction of the antiquated dualistic framework.^34^

### How we are failing people with mental illness

Our default mental framework of dualism creates a perception that mental illness is chronic and difficult to treat or cure. Dualism creates a risk of society entering the slippery slope of rejecting the treatability of mental illness. Given that many physical illnesses share the same degree of chronicity and lack of a cure, it is concerning that such a gap exists between attitudes toward the treatability of mental and physical disorders. Yet, it is rare to see anyone opposing the treatment of chronic and incurable physical health conditions.

This perception of mental illness being untreatable results in stigma, thus causing a delay in seeking help. This results in mental illness remaining undetected and undertreated, which causes a poor prognosis in the long run. Due to reluctance to seek help, patients with mental illness are not benefiting from various screening tests for their physical health, thus resulting in higher morbidity and mortality. There is a synergistic effect of mental and physical comorbidities on IADL functioning, which can result in further health and societal burden.^42^ By adopting a failed and stigmatizing mental framework of dualism, we are doing a disservice to people who develop mental health problems.

## CONCLUSION

Dualism significantly influences our perception of physical and mental health conditions, creating an unbridged gulf between body and mind. Individuals who adhere to dualistic thinking often assume that the mind and body do not interact, leading to the mistaken belief that the brain has no role in creating mental processes. This dualistic mental framework fosters a logical fallacy, resulting in the conclusion that biological treatments—such as psychiatric medications, which act via neurochemicals in the brain—cannot impact mental processes or mental illness.

It is crucial to abandon the mental framework of dualism, which often operates unconsciously and remains unrecognized. Active recognition and addressing of this bias are essential, particularly in the early stages of medical training. Dualism negatively affects our approach to managing the nearly one billion people who develop mental health conditions annually, as well as the treatment of physical morbidity in those with mental illness and those who develop mental illness secondary to chronic physical health conditions.

The future of mental healthcare depends on implementing strategies to counter dualism; however, there are obstacles to these efforts. An institutional shift in priorities and substantial resources are needed for the integration of mental healthcare into acute general hospital settings, for example, even though it is advantageous.^43^ Healthcare professionals used to traditional, dualistic paradigms of treatment may oppose the expansion of liaison psychiatric services and the promotion of shared decision-making.^43^ The integration of mental healthcare with general medical services may also be hampered by budgetary and practical issues.

Addressing dualism and stigma simultaneously presents both opportunities and challenges. Strategies to tackle stigma—such as those that highlight the similarities between mental and physical illness^43^—can help reduce the impact of dualism. However, these efforts must be sustained and supported by ongoing education, policy changes, and public awareness campaigns. Aligning mental health service delivery with mainstream medical services could significantly ameliorate the impact of dualism on the perception of treatability in psychiatry, but this will require concerted efforts across multiple levels of the healthcare system.^43^

Ultimately, while the road to overcoming dualism and its associated stigma is fraught with challenges, the potential benefits for patient care are substantial. By fostering a more integrated and holistic approach to health, we can better serve those with mental health conditions and improve outcomes for all patients.

## Data availability

Not applicable.

## Supplementary material

Not applicable.

## Conflicts of interest

The authors declare that the research was conducted in the absence of any commercial or financial relationships that could be construed as a potential conflict of interest.

## Authors’ contribution

JL, MM, and OW conceived the idea for this opinion manuscript. JL and MM wrote the initial draft of the article. Other authors critically appraised the article and provided input into the final manuscript. All authors approved the final manuscript.

## Funding

This research did not receive any specific grant from funding agencies in the public, commercial, or not-for-profit sectors.
